# The effect of preparation on binding between spatial and non-spatial features of voices in a multitalker setting

**DOI:** 10.1007/s00426-025-02103-6

**Published:** 2025-04-15

**Authors:** Amy Strivens, Aureliu Lavric, Elena Benini, Andrea M. Philipp, Iring Koch

**Affiliations:** 1 Institute for Psychology, RWTH Aachen University, Aachen, Germany; 2 Department of Psychology, Tübingen University, Schleichstraße 4, 72076 Tübingen, Germany; 3 Department of Psychology, University of Exeter, Exeter, UK

## Abstract

Dynamic switching of attention between voices in multitalker situations is often investigated in paradigms that combine selective listening with ‘attention switching’. Participants are presented concurrently with two talkers, a female and a male, and asked to respond to the number spoken by the talker specified on each trial by a cue. A change in the target voice (when compared to listening to the same voice) results in a robust performance ‘switch cost’– which can be reduced substantially by increasing the preparation (cue-stimulus) interval. Using dichotic presentation we asked whether preparation also increases the selectivity for the cued (relevant) voice dimension– gender (in one session) or location (in another session). We examined the interaction between the features of the relevant dimension and features of the irrelevant dimension (which varied independently) as a function of preparation. When the two voices (genders) were heard from the same locations as on the preceding trial, performance was better than when genders swapped locations relative to the previous trial– suggesting ‘binding’ between genders and locations. The key question was whether preparation reduced this binding effect– which would indicate greater dimensional selectivity. We found the opposite– the binding effect was significantly larger when there was more time for preparation. Since preparation reduced the switch cost but increased the binding effect, the results reveal a dissociation between the effect of preparation on the switch cost and on the binding effect. We propose mechanisms by which preparation may enhance the formation of bindings and/or their retrieval.

Since Cherry (1953) posited the seminal “Cocktail Party Problem”, there has been a great deal of interest in investigating how listeners select the target voice in a multitalker setting. To examine how auditory attention is shifted among voices, Koch et al. (2011) adapted the well-established task-cueing version of the task-switching paradigm (Meiran, 1996, for reviews see Kiesel et al., 2010; Koch et al., 2018; Monsell, 2015; Vandierendonck et al., 2010) to develop the cued ‘voice-switching’ paradigm: participants were presented simultaneously with a male voice and a female voice, each saying a number word, and a visual gender cue specified the target voice. Participants had to perform a low/high judgement on the number spoken by the cued target voice. Unlike the more conventional task switching paradigms, this voice-switching paradigm keeps the required categorisation and stimulus-response (S-R) mappings constant, thus isolating the target voice as the only aspect of the task set that could change from one trial to another.

Koch et al. (2011) found a substantial target voice *switch cost*– longer reaction times and higher error rates when the target voice changed compared to when it was repeated. A subsequent study by Lawo et al. (2014) compared the non-spatial (gender) cueing of the target voice (as in Koch et al., 2011) to spatial cueing (by the side/ear where the voice is presented). They found that the switch cost was greater when the target voice was spatially cued than when it was gender-cued. In their third experiment, they also manipulated the preparation interval before the voice compound (cue-stimulus interval; CSI) and found that longer preparation intervals led to a greater reduction in the overall RT in the spatially cued condition than when the target speaker was cued by gender. However, the effect of preparation was not significantly different for switch and repeat trials - preparation did not reduce the switch cost (though it did in some of the subsequent studies, see below). They interpreted these findings as indicating that location-based attentional set has stronger attentional inertia than non-spatial attentional set– thus, the greater benefit of preparation resulted from the extra time required to disengage attention from the previously relevant location (Lawo et al., 2014, see also Koch & Lawo, 2015).

An important aspect of the voice switching paradigm used in the above dichotic listening studies was that the task- (and therefore response-) irrelevant locations of the male and female voices (the side/ear where each gender was heard) varied randomly over trials, independently of the switches or repetitions of features of the cued (target-defining) dimension. Figure 1 illustrates four possibilities for an experimental design where the target-defining dimension is gender^1^.

**Fig. 1:**
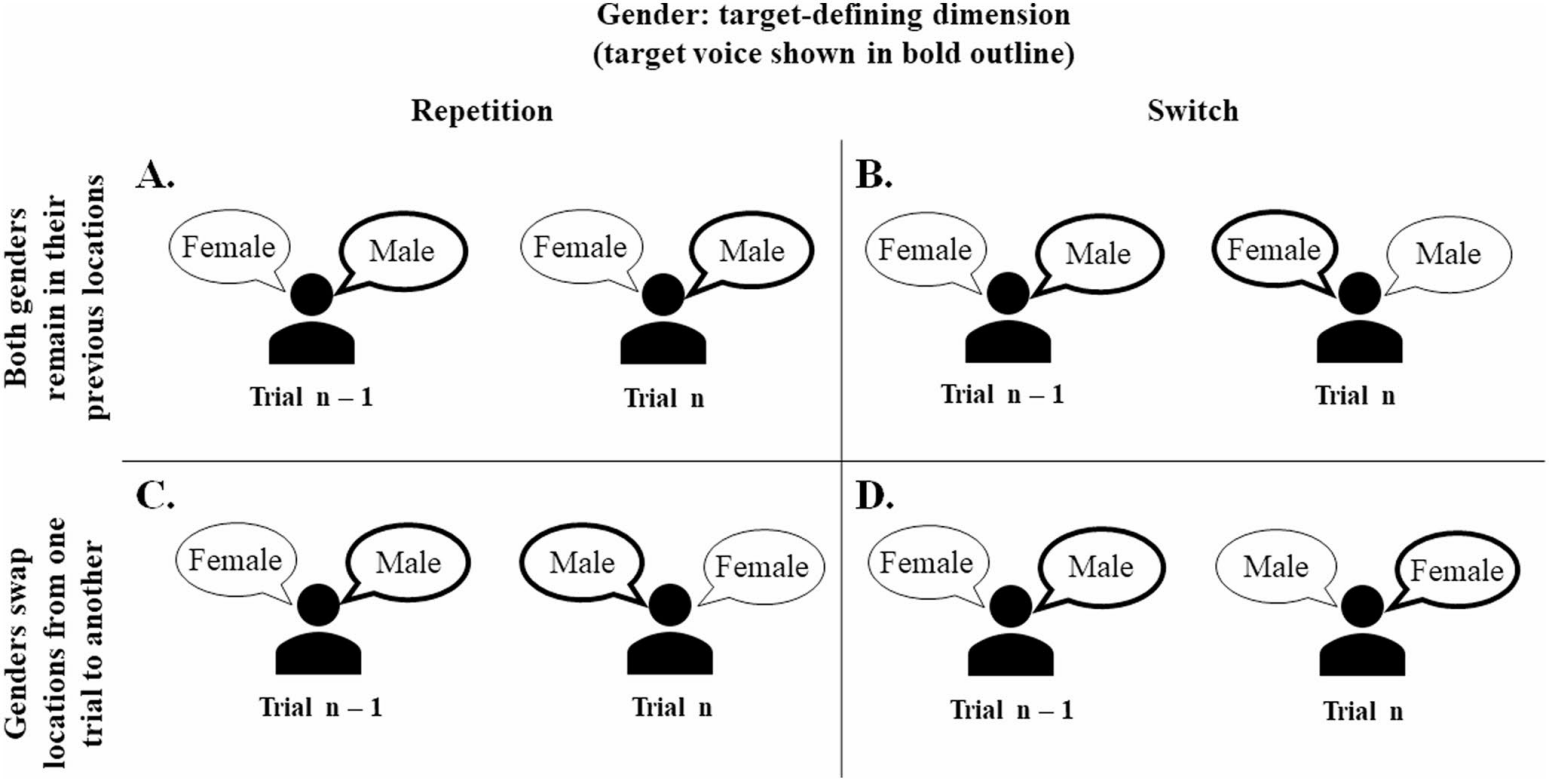
Illustration of a trial-to-trial repetition (panels **A** and **C**) and switch (**B** and **D**) of the feature of the task-relevant dimension (gender) in conditions where the locations of the male voice and female voice stay the same as on the preceding trial (**A** and **B**), and where the locations of the male and female voices swap (**C** and **D**)

In two of the transition possibilities (shown in the two upper panels) the locations of the male and female voices (which we refer to henceforth as *gender-location mappings*) are the same as on the preceding trial. Thus, when the cued gender repeats, the listener must attend to the same gender at the same location as on the previous trial (the gender and the location of the relevant voice both repeat, panel A), whereas when the cued gender switches attention must shift to the gender which was, and still is, at the other location (the gender and location of the relevant voice both switch, panel B). In the remaining two situations (panels C & D) the gender-location mappings swap relative to the preceding trial. Thus, when the cued gender is repeated, the listener hears the relevant voice but on the opposite side relative to trial n-1 (panel C), whereas when the cued gender switches, the listener hears it on the side where they previously heard the other, no-longer relevant, gender (panel D).

According to feature binding approaches, most notably, the *theory of event coding* (Hommel, 2004; see also Frings et al., 2020), whether gender-location mappings are maintained (as in Fig. 1A & B) or not (as in as in Fig. 1C & D) from one trial to the next should have an effect on performance. The central tenet of the theory is that perceptual and action-related attributes (features) of a given trial, including stimulus, response, irrelevant context, etc., are stored together (‘bound’) in an *event file*. When an attribute is repeated on the next trial, this results in automatic retrieval of the whole event file, including the other attributes bound to the attribute which is repeated. This should facilitate performance if the retrieved event file is consistent with the configuration of attributes on the current trial and, conversely, hinder performance if the retrieved event file is inconsistent with the current trial (i.e. a partial mismatch, see Weissman et al., 2023). Applied to the dichotic voice-switching paradigm illustrated in Fig. 1, this account predicts that bindings emerge on each trial between features across voice dimensions (e.g., female-left), resulting in the subsequent retrieval of features of one dimension prompted by the presence of the feature on the other dimension (female → left). This should lead to better performance when the current gender-location mapping is consistent with the binding from trial n-1 (Fig. 1, panels A & B), than when the current gender-location mapping is in conflict with the binding from trial n-1, referred to as a *partial mismatch* in the binding framework (Fig. 1, panels C & D).

Evidence in support of such gender-location binding in selective listening was provided by Koch and Lawo (2014). Although their primary aim was to examine the temporal dissipation of attentional set via a manipulation of the interval between the response and the gender voice cue in a dichotic listening task, the authors also examined the gender switch cost as a function of the change/repetition of the task-irrelevant location (side). They found that “switch costs were reduced when the (entirely task-irrelevant) location of the task-relevant speaker changed, relative to when it was unchanged” (p. 73). Using the convention of Fig. 1, this corresponds to: (B– C) < (D– A), which can arise from better performance when the gender-location mapping is the same as on trial n-1 for gender repetitions (A), and/or gender switches (B), compared to trials where the gender-location mapping is different from trial n-1 (C & D).

Holmes and colleagues (2018) directly compared trials where the gender-location mapping (which they refer to as “configuration”) from the preceding trial is maintained vs. not. Their design adapted the ‘call sign’ paradigm, where several talkers speak phrases like “Ready Baron, go to blue two now”, and the participant executes a response corresponding to the colour-number combination spoken by the talker pre-defined by a call sign (e.g., “Baron”). The authors removed the call sign (leaving only the colour-number combination, e.g., “blue two now”), instead cueing the target voice visually by gender or location. Consistent with the binding framework and with Koch and Lawo’s (2014) findings, they reported faster and more accurate responses when the gender-location mapping (configuration) was repeated over consecutive trials than when it changed; this effect was greater when the voice was cued by gender than by location. However, Holmes et al. did not examine the effect of switching the feature of the relevant dimension and its interaction with the temporal dynamics of gender-location mappings.

Thus, the studies by Koch and Lawo (2014) and Holmes et al. (2018) provided initial evidence suggestive of bindings of non-spatial and spatial features of voices in selective listening situations. However, neither of these studies had feature binding as the primary topic under scrutiny, and the manipulations and analyses relevant to binding were somewhat limited in scope. As already mentioned, Holmes and colleagues did not examine the interaction of binding and the (relevant feature) switch cost. Koch and Lawo (2014) did that, but they did not report in their analyses whether the hypothesised effect of binding is found only (or mainly) for repetitions of features on the cued dimension (better performance in A than in C in Fig. 1), or whether binding has a comparable effect on repetitions and switches (better performance in B than in D in Fig. 1). More importantly, previous voice-cueing studies did not examine whether the effect of binding on performance is affected by advance preparation. The aim of the current study was to examine the effect of preparation on feature binding and on the switch costs.

In the task-set control literature, a manipulation widely believed to reveal the contribution of top-down control to the switch cost is the manipulation of the preparation interval (e.g., Kiesel et al., 2010; Monsell, 2003; Vandierendonck et al., 2010)– typically implemented by varying the CSI (e.g., Meiran, 1996). Increasing the CSI typically results in a substantial reduction in switch cost (e.g., Graham & Lavric, 2021; Meiran, 1996; Monsell & Mizon, 2006; Van’t Wout et al., 2013). Together with converging evidence of switch-related eye-movements (e.g. Longman et al., 2014, 2017) and EEG-derived potentials (Karayanidis et al., 2010; Lavric et al., 2008) during the CSI, the reduction in switch cost with preparation is generally seen as an index of anticipatory top-down control of task-set. Importantly, recent voice-switching studies have also revealed a substantial reduction in the cost of switching the target voice (Lavric & Schmied, 2025; Monsell et al., 2019; Strivens et al., 2024a), especially when the parameters of the paradigm were optimal for encouraging preparation and detecting its effect on performance (Monsell et al., 2019; Strivens et al., 2024a)– an important parameter in this regard is a relatively low probability of a target voice switch (Strivens et al., 2024a).

A key question we ask in the current investigation is whether top-down preparatory control of attentional set modulates the binding effect documented in previous voice-cueing studies. Binding effects are generally seen as emerging from bottom-up, automatic (meaning: involuntary) processes (Frings et al., 2020; Hommel et al., 2001). But– there is a further aspect of automaticity– a process may be involuntary but still susceptible to a degree of top-down control, or it may be invariable– entirely impervious to top-down control. Is the formation or retrieval of bindings between spatial and non-spatial perceptual features of a voice susceptible to top-down control? Since the selection of the relevant voice is based on a single perceptual dimension while another (randomly varying), dimension does not benefit selection, effective attentional control should focus on the relevant dimension and ignore (or even suppress) the encoding of the irrelevant dimension. Thus, increasing the opportunity for preparatory control may result in greater perceptual selectivity, meaning less encoding of the irrelevant dimension, and thus a potential reduction in the formation of bindings of its features to features of the relevant dimension. Hence, we would expect the binding effect on performance to be reduced by preparation, which would be indicated by a significant statistical interaction between CSI and Gender-Location Mapping (our variable that indexes the binding effect; see Method and Results). Alternatively, binding effects may occur at very early (pre-attentional) stages of perceptual encoding and therefore be impervious to the effects of top-down control– in which case preparation and binding (our variables CSI and Gender-Location Mapping) would not be expected to interact.

In addition to distinguishing between the above alternatives, the current study can also address another issue in the voice-switching literature. The above-mentioned study by Holmes et al. (2018) reported (for accuracy, but not RT) a greater effect of maintaining the gender-location mapping when the target voice was specified by gender– suggesting a degree of asymmetry in the strength of the binding between features of the relevant and irrelevant dimensions (stronger when gender is relevant and location is irrelevant than for the converse). We revisit this asymmetry in the current investigation. If the above pattern is present in the current data, we would expect to see a significant interaction between Relevant Dimension (Gender or Location; see Method and Results) and Mapping.

## Method

### Task and materials

**Fig. 2:**
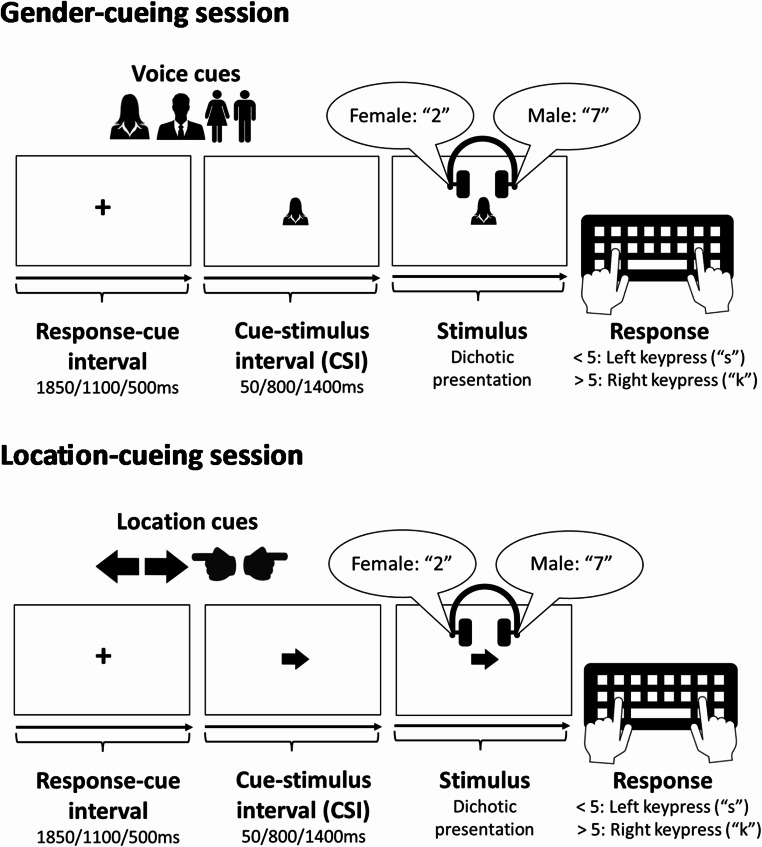
Voice and location cues and the time course of one trial for each session

The experiment was conducted using PsychoPy 3 version v2021.1.4 (e.g. Pierce et al., 2019). Participants were instructed to attend to one of two simultaneous talkers (a male and a female; one in each ear), each saying a number between one and nine, excluding five. The task was to categorise as < or > 5 the number spoken by the voice whose gender or location was cued (in separate sessions) by a pre-stimulus picture cue, and press the ‘s’ key with their left index finger when the number < 5 and the ‘k’ key with their right index finger when the number > 5 (see Fig. 2).

The voice stimuli were numbers spoken in German by two males and two females, recorded in an anechoic chamber at the RWTH Aachen Institute of Medical Acoustics (Oberem & Fels, 2020). The best version of each number was chosen from five samples, adjusted to be 600 ms long and of equal loudness to the other selected utterances. For each of the four male-female pairs, all combinations of the numbers spoken by the two voices (except the combinations where the two talkers spoke the same numbers) were used to create two two-talker compounds; one where the female voice was on the left and the other where it was on the right. Each participant was exposed to two voice pairs– one in the gender-cueing session and one in the location-cueing session, thus ensuring equal familiarity with the voices in the two sessions. Within each session, each of the four voice pairs was used for a quarter of the participants tested (15 out of 60), ensuring that any effects of the experimental manipulations were not limited to one voice pair.

In each session, one of four semantically transparent pictorial cues was displayed centrally (see Fig. 2). In the gender-cueing session, a silhouette or a full-body icon specified the target gender, whereas in the location-cueing session, an arrow and a pointing hand specified the target location. The type of cue (e.g., arrow or hand cue in the location-cueing session) was picked randomly at the beginning of a block and then the two cue types were alternated from one trial to another to avoid immediate cue repetitions, thus unconfounding the voice switch cost from the effect of cue repetition (cf., Koch et al., 2011; Monsell & Mizon, 2006). The cue onset preceded the onset of the voice compound by one of three cue-stimulus intervals (CSIs: 50 ms/800 ms/1400 ms), which was constant within a block but varied over blocks (see below for order of CSIs). The cue remained on the screen until the response or until the 3000 ms response deadline; failure to respond before the deadline or an incorrect keypress led to the central presentation of “Fehler” (“Error”) for 1000 ms. To unconfound the time available for preparation from the time available for the passive decay/dissipation of ‘attentional inertia’, the response-stimulus interval was held at a constant 1900 ms by varying the response-cue interval (during which a central fixation cross was presented) inversely with an increasing CSI: 1850 ms/1100 ms/500 ms.

A script was adapted from Strivens et al. (2024a) to create a unique randomised sequence of trials for each participant and each session. This sequence consisted of three sub-sequences of 240 trials, one for each CSI (each subsequence was then sub-divided into testing blocks, see below). To encourage participants to select the voice specified by the cue, response-incongruent stimuli (where the digits spoken by the two voices required different responses) were presented on the majority of the trials (80%, cf., Monsell et al., 2019). Response-congruent stimuli (where the digits spoken by the two voices required the same response), presented on the remaining 20% of the trials (and excluded from analyses), were only included to deter participants from using the strategy of listening to the same voice on all trials and making the opposite response when the other voice is cued. Given our recent finding that a low probability of a switch is conducive to the reduction in switch cost with preparation (Lavric & Schmied, 2025; Monsell et al., 2019; Strivens et al., 2024a, see Introduction), the cued gender (or location) switched unpredictably on one third of the trials (64 response-incongruent and 16 response-congruent for each CSI), and repeated on the remaining two thirds of trials (128 incongruent and 32 congruent for each CSI). The feature of the task-irrelevant dimension switched unpredictably on 50% of trials orthogonally to (independently of) the switches of the feature of the relevant dimension.

For the (analysed) incongruent trials, all the combinations of CSI x voice gender x location x gender-location mapping transition (same or different from trial n-1) x number spoken by the target (cued) voice were equiprobable for both switch trials (where the relevant feature switched relative to trial n-1) and repeat trials (where the relevant feature was the same as on trial n-1)– each of these combinations occurred once on a switch trial and twice on a repeat trial. Since the number spoken by the target voice required on half of the trials a left-hand response and on half of the trials a right-hand response, the above means that all the combinations of CSI x switch/repeat of relevant feature x voice gender x location x gender-location mapping transition x response were perfectly balanced. For the congruent trials (20% of all trials, not included in analyses), all the combinations of CSI x switch/repeat of relevant feature x voice gender x location x gender-location mapping transition were perfectly balanced, as were all the combinations of CSI x switch/repeat of relevant feature x voice gender x location x response.

Neither of the numbers spoken by the two simultaneous voices were ever repeated on consecutive trials. The three 240-trial sub-sequences (one per CSI) were divided into three blocks of 80 trials plus a start-up (filler) trial excluded from analyses; the voice (or location) on the start-up trial was selected depending on the voice and switch/repeat condition on the subsequent (analysed) trial, whereas the response category and spoken numbers were selected randomly. The 81-trial blocks that used different CSIs were interdigitated by including the 1st block of each CSI, then the 2nd block of each CSI and then the 3rd block of each CSI (whilst preserving the CSI order) e.g. CSI = 50ms-Block1, CSI = 1400ms-Block1, CSI = 800ms-Block1, CSI = 50ms-Block2, CSI = 1400ms-Block2, CSI = 800ms-Block2, etc., resulting in nine blocks of 81 trials (729 trials) in the main part of the experiment. There were six CSI orders rotated over participants^2^ (for a given participant the order of CSIs was the same in the two testing sessions).

### Procedure

The experiment took place across two sessions lasting approximately one hour each with a minimum of 72 h between them. In one session the target voice was cued by gender, whereas in the other the target voice was cued by location/side. Session order was counterbalanced over participants. Each session consisted of three phases: two practice phases and the main phase. The first practice phase included three 16-trial blocks and was designed to familiarise participants with the two voices they would hear throughout the session, the cues to be used in that session and the categorisation task (including the category-response mappings). In each block participants heard only one voice per trial preceded by a gender or location cue at CSI = 500 ms: in the first block they heard only the male voice or only heard a voice on the left (depending on the session), in the second block they heard only the female voice or only heard a voice on the right (depending on the session) and in the third block the two voices were presented in a random order, but still one voice per trial. This was followed by the second practice phase, consisting of three 25-trial practice blocks (one per CSI, starting with the longest CSI and ending with the shortest), where the two voices were presented simultaneously. The structure of each trial in the final phase of practice was the same as in the main phase of the session that followed (see Fig. 2).

### Design

The independent variables were: Relevant Dimension (Gender vs. Location), Switch (switch vs. repetition of the feature of the relevant dimension), Gender-Location Mapping (the locations of the genders– same as on trial n-1 vs. different), and CSI (50ms, 800ms or 1400ms), resulting in a 2 × 2 × 2 × 3 repeated measures design. The dependent variables were RT (ms) and error rate (%).

### Participants

Sixty participants were recruited at RWTH Aachen University. Some were Psychology students who participated in exchange for a course credit, others were not Psychology students who participated without compensation. One participant was excluded from the analysis because their error rate in one session was over three standard deviations above the group mean. Of the 59 participants included in the analysis, 30 were male and 29 female, with a mean age of 24.75 years (SD = 8.92, range = 18–61).

We followed Brysbaert and Stevens’ (2018) recommendations for within-participants experiments based on simulations using data from mega-studies. They recommended a minimum of 1600 observations (over participants and within-participants observations- trials) in the smallest cell of the analysis for detecting an effect with the size d = 0.2 to d = 0.4. The smallest cell of our analysis had 40 observations per participant, meaning that a minimum of 40 participants would be needed to achieve this guideline. With 59 participants and a maximum of 40 analysable trials, the present study had 2360 observations in the smallest cell of our analysis; even assuming a loss ~ 10–15% of the trials due to trials following errors and error trials in the RT analysis, the dataset contained over 2000 observations in the smallest cell– substantially more than recommended by Brysbaert and Stevens and than in both previous studies which reported effects of binding in the context of voice-cueing (Holmes et al., 2018; Koch & Lawo, 2014)^3^.

## Results

**Fig. 3:**
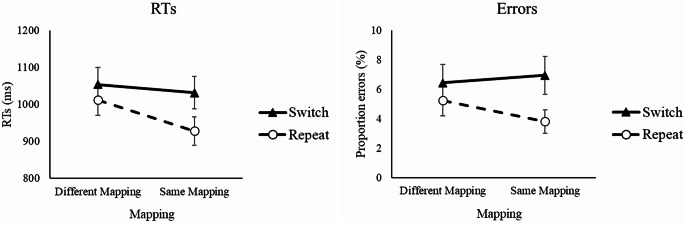
RTs (left panel) and error rates (right panel) as a function of Switch and Mapping. Note. Error bars show 95% confidence intervals around the means

**Fig. 4:**
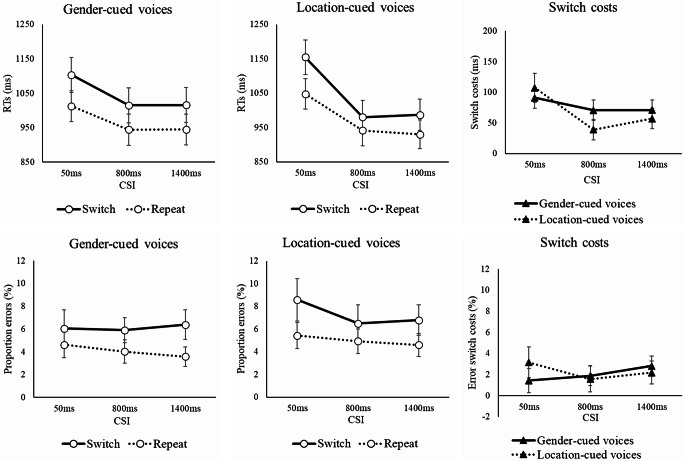
RTs (upper left, and middle panels) and error rates (lower left and middle panels) as a function of CSI, Switch and Relevant Dimension and switch costs (right panels) as a function of CSI and Relevant Dimension. Note. Error bars show 95% confidence intervals around the means

Trials following an error, the first trial of every block and trials with RT < 200 ms were excluded from all analyses; error trials were excluded from RT analyses. As in our previous studies (Monsell et al., 2019; Strivens et al., 2024a, Exp. 1; Strivens et al., 2024b), we limited analyses to incongruent trials, because only on these trials did a correct response require the correct selection of the target voice. Following these exclusions, the participant means were subjected (separately for RTs and errors) to repeated measures ANOVAs with the factors Relevant Dimension (gender vs. location; 2 levels), Switch (of the feature on the relevant dimension, 2), Mapping (of genders to locations– same as on trial n-1 vs. different, 2), and CSI (3). The effects involving factor CSI were Huyhn-Feldt-corrected for sphericity violations where necessary (but uncorrected degrees of freedom are reported). Illustrations of key descriptive statistics are presented in Figs. 3 and 4 and the values are given in Tables 1 and 2.

### Switch cost and binding effect

We started by confirming the switch cost (the cost of switching the target voice). The main effect of Switch was significant for RTs, *F*(1, 58) = 195.18, *p* <.001, η_p_^2^ =.771, and error rates, *F*(1, 58) = 47.96, *p* <.001, η_p_^2^ =.453, revealing a significant switch cost in RTs (73ms) and errors (2.2%), see Fig. 4. The switch cost was significant for both gender-cued [RT, *F*(1, 58) = 132.34, *p* <.001, η_p_^2^ =.695; errors, *F*(1, 58) = 32.69, *p* <.001, η_p_^2^ =.360] and location-cued voices [RT, *F*(1, 58) = 84.71, *p* <.001, η_p_^2^ =.594; errors, *F*(1, 58) = 22.70, *p* <.001, η_p_^2^ =.281], as revealed by a separate ANOVA for each task-relevant dimension.

**Fig. 5:**
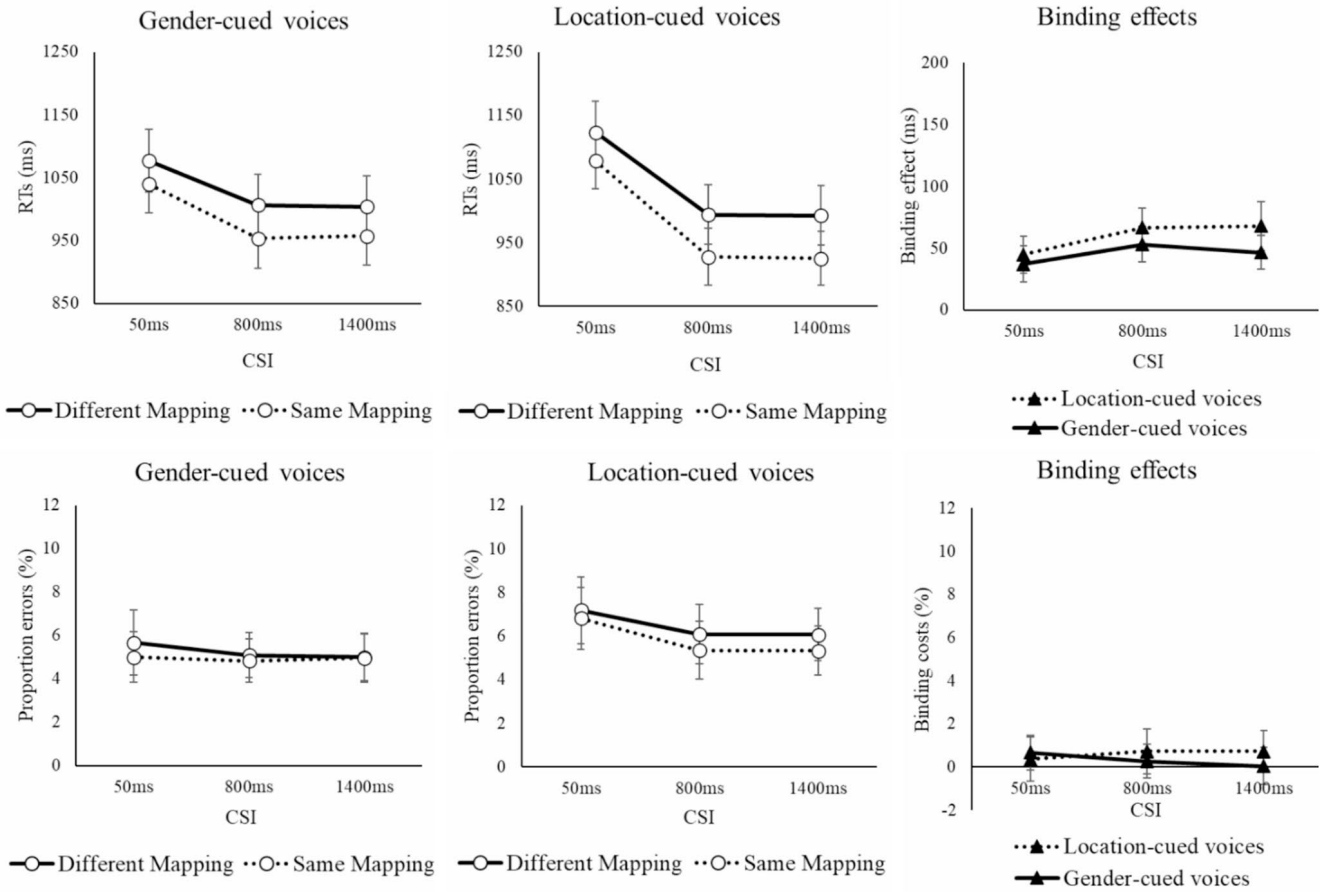
RTs (upper left and middle panels) and error rates (lower left and middle panels) as a function of CSI, Mapping and Relevant Dimension and binding effects (different mapping minus same mapping; right panels). Note. Error bars show 95% confidence intervals around the means

Next we turn to the effect of binding on performance. The main effect of Mapping was significant for RTs, *F*(1, 58) = 118.45, *p* <.001, η_p_^2^ =.671, and errors, *F*(1, 58) = 4.45, *p* =.039, η_p_^2^ =.071, reflecting faster responses and lower error rates when the voices (genders) remained at the same locations as on the previous trial than when the genders swapped locations from the previous trial (980 ms vs. 1033 ms; 5.4% vs. 5.9%), see Fig. 5.

There was a significant interaction between Switch and Mapping for both RTs, *F*(1, 58) = 100.38, *p* <.001, η_p_^2^ =.634, and errors, *F*(1, 58) = 23.01, *p* <.001, η_p_^2^ =.284, reflecting a much larger benefit of keeping the same gender-location mapping as on trial n-1 for repetitions of the target voice than for switches of the target voice, 83 ms vs. 22 ms and 1.4% vs. -0.5% (see Fig. 3), although the Mapping effect was significant even for switches in RTs [ANOVA for repetitions only: RT, *F*(1, 58) = 190.33, *p* <.001, η_p_^2^ =.766; errors, *F*(1, 58) = 23.01, *p* <.001, η_p_^2^ =.284; ANOVA for switches only: RT, *F*(1, 58) = 16.29, *p* <.001, η_p_^2^ =.219; errors, *F*(1, 58) = 2.17, *p* =.146, η_p_^2^ =.036].

There was a significant Mapping x Relevant Dimension interaction for RTs, *F*(1, 58) = 5.20, *p* =.026, η_p_^2^ =.082 (but not errors, *F* < 1). This interaction reflected a greater RT benefit of encountering the same mapping as on the previous trial when location was the relevant dimension than when gender was relevant (60 vs. 46 ms; see rightmost panel of Fig. 5). Separate RT ANOVAs by task-relevant dimension found the main effect of Mapping to be significant for each dimension when it was task-relevant (location, *F*(1, 58) = 97.49, *p* <.001, η_p_^2^ =.627; gender, *F*(1, 58) = 70.07, *p* <.001, η_p_^2^ =.547).

### Effects of preparation on binding and the switch cost

Of central importance to the current investigation is whether preparation (manipulated via factor CSI) influences the binding effect, as reflected in the effect of factor Mapping. These factors indeed interacted for RTs (but not errors, *F* < 1), *F*(2, 116) = 4.69, *p* =.014, η_p_^2^ =.075, which reflected a larger binding effect (benefit of keeping the same gender-location mapping as on trial n-1) for the two longer CSIs of 800 ms and 1400 ms (60 ms and 57 ms respectively) than for the short CSI of 50 ms (41 ms).

Prolonging the CSI resulted in better overall performance averaging over other factors (1080 ms, 970 ms, 970 ms; 6.2%, 5.3%, 5.3%), as shown by the significant main effect of CSI for RT, *F*(2, 116) = 145.89, *p* <.001, η_p_^2^ =.716, and errors, *F*(2, 116) = 6.68, *p* =.002, η_p_^2^ =.103). A longer CSI also significantly reduced the switch cost for RTs (99ms, 55ms, 64ms, in the order of increasing CSI), as indicated by the significant CSI x Switch interaction, *F*(2, 116) = 21.11, *p* <.001, η_p_^2^ =.267, though this interaction did not approach significance in the error rates, *F*(2, 116) = 1.44, *p* =.240, η_p_^2^ =.024. The reduction in switch cost with preparation was steeper when the target voice was cued by location than when it was cued by gender (see Fig. 4), as indicated by the significant CSI x Switch x Relevant Dimension interaction for RTs, *F*(2, 116) = 5.50, *p* =.005, η_p_^2^ =.087 (for errors, this interaction narrowly failed to reach significance, *F*(2, 116) = 3.04, *p* =.053, η_p_^2^ =.050). Still, the switch costs were reduced significantly with preparation for both location-cueing and gender-cueing conditions, as indicated by the Switch x CSI interactions in the separate follow-up ANOVAs for gender cueing, *F*(2, 116) = 3.20, *p* =.044, η_p_^2^ =.052, and location cueing, *F*(2, 116) = 19.93, *p* <.001, η_p_^2^ =.256.

We also examined whether binding may modulate the reduction in switch cost with preparation. The three-way interaction between CSI, Switch and Mapping did not reach significance for either RTs, *F*(2, 116) = 2.35, *p* =.100, η_p_^2^ =.039, or error rates, *F* < 1.

### Other effects of relevant dimension

There was a significant main effect of Relevant Dimension for errors, *F*(1, 58) = 8.82, *p* =.004, η_p_^2^ =.132 (but not RTs; *F* < 1), reflecting a higher error rate in the location cueing condition (6.1%) than the gender cueing condition (5.1%). There was also a significant interaction between CSI and Relevant Dimension, *F*(2, 116) = 19.27, *p* <.001, η_p_^2^ =.249, with the reduction in overall RTs across CSI being greater in the location-cued condition (142 ms) than the gender-cued condition (77 ms); this interaction was not significant in the error rates, *F*(2, 116) = 1.87, *p* =.159, η_p_^2^ =.031.

**Table 1:** Mean RTs (ms) as a function of relevant dimension, CSI, switch and mapping

Relevant Dimension	Mapping	Switch							
		Switch				Repeat			
		Same Mapping		Different Mapping		Same Mapping		Different Mapping	
	**CSI**	**M**	**SD**	**M**	**SD**	**M**	**SD**	**M**	**SD**
**Gender**	**50ms**	1102	196	1105	215	1048	185	977	169
	**800ms**	1000	201	1030	205	906	178	983	183
	**1400ms**	1003	190	1029	216	911	180	979	178
**Location**	**50ms**	1154	196	1156	209	1004	166	1091	188
	**800ms**	964	196	997	192	891	162	991	190
	**1400ms**	968	183	1006	189	881	159	979	186

**Table 2:** Error rates (%) as a function of relevant dimension, CSI, switch and mapping

Relevant Dimension	Mapping	Switch							
		Switch				Repeat			
		Same Mapping		Different Mapping		Same Mapping		Different Mapping	
	**CSI**	**M**	**SD**	**M**	**SD**	**M**	**SD**	**M**	**SD**
**Gender**	**50ms**	6.02	6.27	6.08	7.30	3.98	4.27	5.26	5.37
	**800ms**	6.16	5.13	5.65	5.29	3.50	4.21	4.54	4.80
	**1400ms**	6.89	6.56	5.87	5.18	3.03	3.49	4.12	4.32
**Location**	**50ms**	9.04	8.15	8.11	8.37	4.59	4.78	6.25	5.27
	**800ms**	6.83	7.59	6.16	6.95	3.86	4.20	5.99	5.08
	**1400ms**	6.75	6.26	6.82	5.94	3.90	4.11	5.31	4.80

### Effects of binding perceptual voice dimensions and responses

Binding research often investigates the binding between stimulus attributes and responses (e.g. Hommel, 1998; Hommel & Frings, 2020; Janczyk et al., 2023). Hence, we performed an exploratory analysis to test for evidence of three-way binding between features of the target dimension of the voices, features of the non-target dimension of the voices, and responses. We submitted the data to repeated measures ANOVAs for RTs and errors, with the factors Switch (of feature on the relevant dimension), Mapping (of gender to locations: same as on trial n-1 vs. not), and Response Transition (switch vs. repetition); we had to collapse across CSI levels to ensure a sufficient number of observations per participant.

We focus on the interaction between Mapping and Response Transition^4^– which was significant for RTs, *F*(1, 58) = 9.55, *p* =.003, η_p_^2^ =.141, but not for errors (*F* < 1). The effect of keeping the same gender-location mapping as on trial n-1 was greater when the response was also the same as on trial n-1 than when the response changed (61 ms vs. 44 ms)– which seems to suggest additional binding between perceptual features and responses. However, we caution that our study was not designed to investigate bindings that include the response^5^.

## Discussion

The present study investigated whether preparing to listen to one of two simultaneous dichotic voices influences the bindings between task-relevant and task-irrelevant perceptual features of voices. To this end, we employed the voice-switching paradigm of Koch et al. (2011), which combines selective listening in a multitalker setting with aspects of task-switching (see Introduction). We cued at three CSIs either the gender of the target voice or its location whilst the task-irrelevant dimension varied orthogonally, which allowed us to examine the effect of preparation on binding of task-relevant and irrelevant features in a selective listening setting. We reasoned that if preparation increases attentional/perceptual selectivity, then it should reduce the encoding of the features of the task-irrelevant dimension– which in turn should result in reduced effects of binding with a longer CSI.

Our analyses revealed the expected (based on prior research, see Introduction) benefit to performance of maintaining the gender-to-location mappings from one trial to the next– indicative of binding of gender and location features. This was the case both when gender was task-relevant (as in Koch & Lawo, 2014) and when location was task-relevant (confirming Holmes et al., 2018). We extended the findings of Koch and Lawo (2014) and Holmes et al. (2018) by showing that this binding effect was greater on repetition trials than on switch trials. This means that the difference between full repetition trials (panel A of Fig. 1) and partial repetition trials (panel C) was larger than the difference between full switches (panel D) partial repetitions (panel B), consistent with the event coding framework (e.g., Frings et al., 2020; Hommel, 2004; Weissman et al., 2023).

Crucially, our results do not support the notion that preparatory attentional tuning to a non-spatial or spatial feature of a voice leads to greater dimensional selectivity– the effect of binding on performance did not reduce with preparation. On the contrary, a longer preparation interval (CSI) resulted in a significantly larger binding effect. One may ask whether the reason that preparation did not increase dimensional selectivity was that participants did not take advantage of the opportunity to exert top-down attentional control during the longer preparation interval in blocks with a long CSI. However, this interpretation is invalidated by our observation, for both gender-cued and location-cued voices, of a robust reduction in switch cost with a longer CSI– widely regarded as an index of top-down control (see Introduction).

What process(es) may be responsible for the observed increase in the binding effect when the CSI was longer? One likely possibility is that by virtue of retrieving in advance the feature on the task-relevant dimension (e.g., the fundamental frequency of the female voice when the female gender is cued), preparatory attentional control may also retrieve the feature on the task-irrelevant dimension that was last bound to the task-relevant feature (e.g., ‘left’ if the female voice was last heard on the left). This interpretation predicts stronger retrieval of the previously bound task-irrelevant feature at longer preparation intervals and is consistent with recent studies by Seibold et al. (2018, 2019). These studies explored the binding between a target stimulus location and a judgement task in a paradigm that combined voice switching and task switching. In their Experiment 3, where they manipulated the CSI, they found that a longer preparation interval led to a stronger interaction between judgement switches and attention switches. Seibold and colleagues concluded that a longer CSI provided more time to retrieve an existing binding. Furthermore, previous task switching research investigating whether a task-irrelevant context is bound to the task and response found that presenting the task-irrelevant context before the stimulus leads to a stronger binding effect than their simultaneous presentation (Benini et al., 2023a). This effect was again attributed to the availability of time for the binding to be retrieved before the stimulus onset in the non-simultaneous condition (see also Benini et al., 2023b). While these studies did not examine binding between perceptual features of the stimulus, the general notion that extra time can facilitate the retrieval of already formed bindings applies to our results.

Another possibility for explaining the observed increase in the binding effect with a longer CSI is that preparation may facilitate the initial process of binding formation. Although the feature on the task-irrelevant dimension is not available in advance of stimulus onset (only the feature on the task-relevant dimension is cued), preparatory tuning for the cued feature may result, following stimulus onset, in better encoding of the task-irrelevant feature physically related to the cued (and prepared, when the CSI is long) task-relevant feature, and hence in a stronger binding between the two. For example, if a spatial cue specifies the left side as target location in advance of the voice compound which has the female voice on the left, this may lead, after stimulus onset, not only to spatial selection of the auditory signal heard in the left ear, but also to better encoding of the frequency range corresponding to the female voice than of the frequency range corresponding to the male voice. This boost in perceptual encoding not only for the task-relevant feature (left location in this example), but also for the physically related task-irrelevant feature (frequency range of female voice) may result in stronger binding between the features. According to this account, it is the initial binding between features of the current trial that is strengthened by longer preparation.

It is important to note that the account of the influence of preparation on binding formation and the account presented earlier where preparation benefits retrieval of already formed bindings are not mutually exclusive– both may be at play. Our results are also compatible with an account in terms of voices being selected and encoded as multidimensional ‘auditory objects’– which favours performance in conditions where the relationship/mapping between dimensions is maintained (e.g., Best et al., 2008, 2010; Holmes et al., 2018: Shinn-Cunningham, 2008). However, we note that because this account is concerned exclusively with the integration of perceptual features, it is not obvious how it would account for our additional finding that the benefit of maintaining the gender-location mappings over consecutive trials was greater when the response was also repeated from one trial to the next. Accounts in terms of binding that allow for the integration of perceptual and non-perceptual features would naturally accommodate this finding.

Our finding of an increase in the binding effect with preparation may also be relevant for a long-standing debate in the task-switching literature regarding the source of the switch cost. In particular, some accounts have proposed that the switch cost can be explained (nearly) entirely by differences in episodic feature priming between task switches and task repetitions (e.g., Logan & Bundesen, 2003; Schmidt et al. 2016, 2020), challenging the notion that a (substantial portion) of the switch cost reflects top-down task-set control. The more recent of these accounts (Schmidt et al. 2016, 2020) have specifically focused on the role of integration between features in explaining the task switch cost. Our finding of a ‘double dissociation’ between the effect of preparation on the switch cost and on the binding effect– a reduction in the former and an increase in the latter with a longer CSI, suggests that a substantial portion of the switch cost (at least in the confines of the present selective listening paradigm) is not easily attributable to feature integration, and that the source of the switch cost reduced by preparation is more likely top-down control of task-set.

We now turn to our finding that the effect of binding was significantly larger when location was the task-relevant dimension and gender was task-irrelevant. To explain this, we consider real-life selective listening scenarios and assume that in such scenarios, it is more common to listen (attend) to a talker (whatever their location) than a location (whatever the talker at that location). This is particularly obvious in settings where spatial location is not a reliable clue to the identity of the talker (e.g., densely packed crowd, online meeting with multiple attendees, etc.). If this assumption is indeed correct, then it may explain why task-irrelevant non-spatial features which identify the talker may be encoded more strongly than the irrelevant location of the voice. However, this can only be a tentative interpretation. As mentioned in the Introduction, Holmes et al. (2018) reported a larger binding effect when gender was the relevant dimension– the opposite of what we found. Future research will have to determine which of these empirical effects (if any) stands, before further interpretations are put forward.

The primary aim of the current study was to examine whether preparatory attentional tuning of auditory attention to a non-spatial or spatial feature of a voice reduces the interaction (binding) of that dimension with a task-irrelevant perceptual dimension, thus increasing dimensional selectivity. Our results reveal quite the opposite– that, intriguingly, preparation resulted in a stronger binding between features on the task-relevant dimension and features on the task-irrelevant dimension. This finding suggests that preparation benefits the retrieval and/or the formation of bindings– but future research will need to clarify which of these processes is (more) facilitated by preparation.

## Appendix

Table of complete inferential statistics

**Table:** 

Analysis	Effect	F	df	p	Ƞ_p_^2^
RT: Relevant Dimension x CSI x Switch x Mapping	Relevant Dimension	< 0.01	1, 58	.967	< .001
	CSI	145.89	2, 116	< .001	.716
	Switch	195.18	1, 58	< .001	.771
	Mapping	118.45	1, 58	< .001	.671
	Relevant Dimension x CSI	19.27	2, 116	< .001	.249
	Relevant Dimension x Switch	1.06	1, 58	.309	.018
	CSI x Switch	21.11	2, 116	< .001	.267
	Relevant Dimension x CSI x Switch	5.50	2, 116	.005	.087
	Relevant Dimension x Mapping	5.20	1, 58	.026	.082
	CSI x Mapping	4.69	2, 116	.014	.075
	Relevant Dimension x CSI x Mapping	0.50	2, 116	.608	.009
	Switch x Mapping	100.39	1, 58	< .001	.634
	Relevant Dimension x Switch x Mapping	1.72	1, 58	.195	.029
	CSI x Switch x Mapping	2.35	2, 116	.100	.039
	Relevant Dimension x CSI x Switch x Mapping	0.02	2, 116	.985	< .001
RT Gender-cued: CSI x Switch x Mapping	CSI	65.64	2, 116	< .001	.531
	Switch	132.34	1, 58	< .001	.695
	Mapping	70.07	1, 58	< .001	.547
	CSI x Switch	3.20	2, 116	.044	.052
	CSI x Mapping	1.83	2, 116	.167	.031
	Switch x Mapping	46.72	1, 58	< .001	.446
	CSI x Switch x Mapping	1.18	2, 116	.310	.020
RT Location-cued: CSI x Switch x Mapping	CSI	112.67	2, 116	< .001	.660
	Switch	84.71	1, 58	< .001	.594
	Mapping	97.49	1, 58	< .001	.627
	CSI x Switch	19.93	2, 116	< .001	.256
	CSI x Mapping	2.93	2, 116	.059	.048
	Switch x Mapping	44.94	1, 58	< .001	.437
	CSI x Switch x Mapping	1.17	2, 116	.314	.020
RT switch trials: Relevant Dimension x CSI x Mapping	Relevant Dimension	0.06	1, 58	.809	.001
	CSI	131.19	2, 116	< .001	.693
	Mapping	16.29	1, 58	< .001	.219
	Relevant Dimension x CSI	18.92	2, 116	< .001	.246
	Relevant Dimension x Mapping	0.37	1, 58	.546	.006
	CSI x Mapping	5.09	2, 116	.010	.081
	Relevant Dimension x CSI x Mapping	0.19	2, 116	.825	.003
RT repetition trials: Relevant Dimension x CSI x Mapping	Relevant Dimension	0.11	1, 58	.737	.002
	CSI	106.85	2, 116	< .001	.648
	Mapping	190.33	1, 58	< .001	.766
	Relevant Dimension x CSI	9.56	2, 116	< .001	.142
	Relevant Dimension x Mapping	5.39	1, 58	.024	.085
	CSI x Mapping	0.64	2, 116	.528	.011
	Relevant Dimension x CSI x Mapping	0.47	2, 116	.629	.008
RT: Switch x Mapping x Response Transition	Switch	194.18	1, 58	< .001	.770
	Mapping	123.71	1, 58	< .001	.681
	Response Transition	38.69	1, 58	< .001	.400
	Switch x Mapping	94.48	1, 58	< .001	.620
	Switch x Response Transition	37.57	1, 58	< .001	.393
	Mapping x Response Transition	9.55	1, 58	.003	.141
	Switch x Mapping x Response Transition	8.60	1, 58	.005	.129
RT: Stimulus Location x Response Location	Stimulus Location	29.37	1, 58	< .001	.336
	Response Location	4.07	1, 58	.048	.066
	Stimulus Location x Response Location	47.26	1, 58	< .001	.449
Error: Relevant Dimension x CSI x Switch x Mapping	Relevant Dimension	8.82	1, 58	.004	.132
	CSI	6.68	2, 116	.002	.103
	Switch	47.96	1, 58	< .001	.453
	Mapping	4.45	1, 58	.039	.071
	Relevant Dimension x CSI	1.87	2, 116	.159	.031
	Relevant Dimension x Switch	0.21	1, 58	.650	.004
	CSI x Switch	1.44	2, 116	.240	.024
	Relevant Dimension x CSI x Switch	3.04	2, 116	.053	.050
	Relevant Dimension x Mapping	0.49	1, 58	.488	.008
	CSI x Mapping	0.05	2, 116	.954	.001
	Relevant Dimension x CSI x Mapping	0.74	2, 116	.470	.013
	Switch x Mapping	23.01	1, 58	< .001	.284
	Relevant Dimension x Switch x Mapping	0.61	1, 58	.439	.010
	CSI x Switch x Mapping	0.09	2, 116	.919	.001
	Relevant Dimension x CSI x Switch x Mapping	0.75	2, 116	.470	.013
Errors Gender-cued: CSI x Switch x Mapping	CSI	0.80	2, 116	.446	.014
	Switch	32.69	1, 58	< .001	.360
	Mapping	1.51	1, 58	.224	.025
	CSI x Switch	2.35	2, 116	.107	.039
	CSI x Mapping	0.66	2, 116	.518	.011
	Switch x Mapping	9.20	1, 58	.004	.137
	CSI x Switch x Mapping	0.23	2, 116	.794	.004
Errors Location-cued: CSI x Switch x Mapping	CSI	6.18	2, 116	.003	.096
	Switch	22.70	1, 58	< .001	.281
	Mapping	3.31	1, 58	.074	.054
	CSI x Switch	2.31	2, 116	.104	.038
	CSI x Mapping	0.20	2, 116	.812	.003
	Switch x Mapping	14.29	1, 58	< .001	.198
	CSI x Switch x Mapping	0.51	2, 116	.585	.009
Error switch trials: Relevant Dimension x CSI x Mapping	Relevant Dimension	5.85	1, 58	.019	.092
	CSI	3.49	2, 116	.037	.057
	Mapping	2.17	1, 58	.146	.036
	Relevant Dimension x CSI	2.89	2, 116	.062	.048
	Relevant Dimension x Mapping	< 0.01	1, 58	.972	< .001
	CSI x Mapping	0.02	2, 116	.982	< .001
	Relevant Dimension x CSI x Mapping	0.87	2, 116	.414	.015
Error repetition trials: Relevant Dimension x CSI x Mapping	Relevant Dimension	4.72	1, 58	.034	.075
	CSI	6.94	2, 116	.001	.107
	Mapping	32.78	1, 58	< .001	.361
	Relevant Dimension x CSI	0.14	2, 116	.866	.002
	Relevant Dimension x Mapping	1.79	1, 58	.186	.030
	CSI x Mapping	0.21	2, 116	.806	.004
	Relevant Dimension x CSI x Mapping	0.41	2, 116	.665	.007
Errors: Switch x Mapping x Response Transition	Switch	47.16	1, 58	< .001	.448
	Mapping	5.03	1, 58	.029	.080
	Response Transition	58.60	1, 58	< .001	.503
	Switch x Mapping	22.35	1, 58	< .001	.278
	Switch x Response Transition	35.27	1, 58	< .001	.378
	Mapping x Response Transition	0.75	1, 58	.391	.013
	Switch x Mapping x Response Transition	2.32	1, 58	.133	.038
Errors: Stimulus Location x Response Location	Stimulus Location	14.50	1, 58	< .001	.200
	Response Location	0.16	1, 58	.690	.003
	Stimulus Location x Response Location	45.64	1, 58	< .001	.440

## Data Availability

The materials used in the study, the data obtained in the study, and the outputs of the statistical analyses are available for open access in the PsychArchives repository; 10.23668/psycharchives.16175.

## Footnotes

1 Female and male voices are likely distinguished based on a number of perceptual attributes– such as fundamental frequency and vocal tract length. For simplicity, to distinguish gender-cued selection from spatial selection, we refer collectively to the attributes that underlie gender-cued selection as the ‘gender dimension’.

2 The six CSI orders were: 50-800-1400; 50-1400-800; 800-50-1400; 800-1400-50; 1400-50-800; 1400-800-50.

3 As a further check of the experiment’s power, we performed a post hoc test of achieved power in G*Power (Faul et al., 2007). We focused on our main CSI x Mapping interaction of interest (3 × 2). With alpha set to 0.05 and assuming a medium effect size of d = 0.5, our 59 participants would give us an achieved power of 0.93.

4 This analysis also revealed a significant main effect of Response Transition for RTs, *F*(1, 58) = 38.69, *p* <.001, ηp2 =.400, and error rates, *F*(1, 58) = 58.60, *p* <.001, ηp2 =.503, but these reflected opposite effects for the two measures– longer RTs, but fewer errors, for response repetitions (1017 ms; 4.5%) than switches (995 ms; 6.5%). Switch and Response Transition interacted significantly, but again in opposite directions, for RTs, *F*(1, 58) = 37.57, *p* <.001, ηp2 =.393, and error rates, *F*(1, 58) = 35.27, *p* <.001, ηp2 =.378. The switch cost was larger in RTs, but smaller for errors (90 ms, 0.7%), when the response repeated than when it changed (55 ms; 3.6%).

5 Following the suggestion of a reviewer, we also examined whether the location of the cued (to-be-attended) voice interacted with the location of the response. To test this, we used a 2 (Stimulus Location; Left vs. Right) x 2 (Response Location; Left vs. Right) repeated measures ANOVA. RTs were 29 ms shorter for trials where the cued voice and response hand were on the same side than for trials where the response was made on the opposite side from the cued voice, *F*(1, 58) = 47.26, *p* <.001, ηp2 =.449; however, in the error rate a significant difference of -1.7% in the opposite direction was observed, *F*(1, 58) = 45.64, *p* <.001, ηp2 =.440. These opposing patterns in RT and error rate are difficult to interpret and may be indicative of a speed-accuracy trade-off.
